# Characteristics of Emergency Department Patients Referred to an Undiagnosed Mass Clinic

**DOI:** 10.5811/westjem.41793

**Published:** 2025-09-25

**Authors:** Brittany Beel, Ryan T. McKenna, Jesse W St Clair, Joan M. Irizarry-Alvarado, Greg E. Coltvet, Johnathan M. Sheele

**Affiliations:** * Mayo Clinic, Department of Emergency Medicine, Jacksonville, Florida; † Mayo Clinic, Division of General Internal Medicine, Jacksonville Florida

## Abstract

**Introduction:**

The emergency department (ED) serves as an entry point to the healthcare system for many patients, and the increased use of advanced imaging has resulted in identification of masses of unclear significance. We describe patients presenting to an ED who were referred to an undiagnosed mass clinic (UMC).

**Methods:**

We performed a retrospective observational cohort study of patients ≥16 years of age presenting to Mayo Clinic in Jacksonville, Florida, from October 31, 2018–March 31, 2023, who were referred to the UMC.

**Results:**

There were 116 patients referred to the UMC with a median of 3.5 days from ED encounter to clinic date and a median of 14.5 days from ED encounter to biopsy. Using an analytic tool in the electronic health record, we estimated that of 16,872 patients, 116 (0.69%) Mayo Clinic Florida (MCF) ED patients ≥18 years of age who received computed tomography and were discharged from the ED were referred to the UMC. Ultimately, 35 of 65 patients (53.8%) seen in the UMC received a cancer diagnosis.

**Conclusion:**

Our study shows a viable care path from ED encounter to undiagnosed mass clinic. Further research is needed to ensure timely transitions of care for patients who are uninsured or out of network.

## INTRODUCTION

Since its inception, the role of the emergency department (ED) has expanded from emergency and acute, unscheduled episodic care to becoming the gateway to the hospital and broader healthcare system for both established and new patients.^1^ For many of these patients, the ED is their first encounter with the healthcare system, serving as a bridge for temporizing care and obtaining referrals while awaiting specialty care. With the increasing number of advanced imaging studies, such as computed tomography (CT) and magnetic resonance imaging being performed in the ED, it is not uncommon for patients to have masses of unclear significance found incidentally during their ED evaluation.^2^,^3^ Up to 50% of common cancers are diagnosed in the ED.^4^

Delays in treatment of as few as four weeks are associated with increased mortality in certain types of cancer and disproportionally affect patients from minority and low socioeconomic populations.^5^,^6^ Diagnosis of a potential new malignancy can be devastating, and patients need close follow-up to determine the need for further imaging, biopsy, staging, and treatment. Previous research has demonstrated the feasibility of transitioning care of patients with suspicious masses from the ED to the outpatient setting.^7^,^8^ In this paper, we describe our experience and the characteristics of patients presenting to the ED at Mayo Clinic in Jacksonville, Florida (MCF), who were referred to an undiagnosed mass clinic (UMC).

## METHODS

This was a quality improvement project that was approved by the institutional review board (IRB) after data was collected. We performed a retrospective chart review after the IRB deemed this study exempt. We collected data through manual chart abstraction from ED referrals made to the UMC at MCF, from the time the clinic opened on October 31, 2018, through March 31, 2023, for patients ≥16 years of age, until over 100 referrals were reached. The following data were obtained: patient demographics; ED chief complaints; ED diagnoses; ED and UMC arrival dates; documented reasons for loss to follow-up; and whether the patient had a new diagnosis of cancer, was receiving care at Mayo Clinic, or was deceased. We removed from the analysis any patient who had followed up in the UMC but was missing data for the variable of interest. This occurred in four instances. We followed all elements of retrospective chart review with one exception: An experienced ED administrator who was not blinded to the study objectives abstracted the data.^9^

## RESULTS

During the study period, there were 141,316 patient visits to the ED. A total of 116 patients were referred to the UMC by 28 emergency clinicians. Using the SlicerDicer data visualization and reporting program in our electronic health record (Epic Systems Corporation, Verona, WI), we estimated that 116/16,872 (0.69%) MCF ED patients ≥ 18 years of age who received a CT and were discharged from the ED were referred to the UMC. The clinicians made a median (range) of three (1–28) referrals. The median (IQR) age for patients referred to the UMC was 62.5 (48.7–71.0) years, of whom 92 (79.3%) were White, and 63 (54.3%) were women. Patients were seen in the UMC a median (IQR) of 3.5 (22.3) days after the ED encounter. Among those patients referred to the UMC who were found to have a new diagnosis of cancer, the median (IQR) time from ED encounter to UMC appointment was four (2.3–5.8) days; from UMC appointment to biopsy was 7.5 (3.5–14.3) days; and from ED encounter to biopsy was 14.5 (7.0–19.3) days.

The most common chief complaints referred to the UMC were abdominal pain, headache, and chest or chest wall pain (Table). Among the 65 patients seen in the UMC, 35 (53.8%) received a diagnosis of cancer. Of those, 31 patients (88.6%) had a new cancer diagnosis, while for one (2.9%) patient the cancer diagnosis was not new and for three (8.6%) it was unclear whether it was a new cancer diagnosis. Among those with a new diagnosis of cancer, 20 (64.5%) were still alive at the time of publication of this paper and six (19.4%) had died; it is unclear whether the other five (16.1%) were still alive. Also, 23 patients (74.2%) with new cancer diagnoses were receiving cancer treatment at Mayo Clinic.

Fifty-one (44.0%) referrals to the UMC resulted in the patient not being seen. Reasons for this include condition was out of the scope of the clinic (16); insurance was out of network (8); patients opted out who were self-pay or uninsured (4); the patient had other specialist follow-up or referral to another clinic (9), declined or canceled the UMC appointment (5), was not reachable (6), was hospitalized (2), or did not show for appointment (1). Of the five patients who died, three declined or canceled the UMC appointment and two had other specialist follow-ups or were referred to another clinic.

**Population Health Research Capsule**

What do we already know about this issue?

*Masses of undetermined significance are often incidentally found in the emergency department (ED). Delays in evaluation and treatment can lead to increased mortality*.

What was the research question?

*Is referring ED patients to a clinic a viable method for transitions of care, and what gaps and opportunities exist?*

What was the major finding of the study?

*Of the 65 patients seen in the undiagnosed mass clinic, 35 (53.8%) were diagnosed with cancer, of whom 31 (88.6%) had a new cancer diagnosis*.

How does this improve population health?

*We describe an alternative to admitting stable patients with an undiagnosed mass, instead referring them to an outpatient clinic for expedited evaluation*.

## DISCUSSION

This study shows the viability of transitioning the care of patients seen in the ED with undiagnosed masses to an outpatient clinic such as the UMC at our institution, which can expedite workup. Previous studies have described the feasibility of accelerated coordination-of-care pathways from the ED and outpatient settings.^7^–^9^ A substantial portion (54%) of our study population received a new diagnosis of cancer, comparable to a similar ED pathway at a different institution (53%).^8^ Patients seen in a primary care setting had a lower percentage of incidental cancer findings (4%) than our patient population.^11^ In a retrospective study conducted at a Level I trauma center, only 15% of incidental findings were considered clinically concerning by the study authors, which included masses suggestive of primary malignancy and potential metastatic lesions.^12^ This difference is likely due to the baseline characteristics of patient populations presenting to each ED—one is a trauma center while ours is a complex, comprehensive medical center. A previous study at our institution from multiple referral sources found that most patients were seen within nine days from initial contact.^9^ Demographics were similar in this cohort; however, the mean time to follow-up in our study of exclusively ED patients was 3.5 days, instead of nine days, due to an established clinic scheduling protocol for expediting ED patient follow-up. Rapid follow-up is key during this period of uncertainty, fear, and anxiety for patients.

## LIMITATIONS

This was a single-center, retrospective chart review at a complex, comprehensive medical center with limited racial diversity. The predominance of the White population referred to the UMC reflects the socioeconomic population that visits the ED and clinics at MCF. Our institution’s insurance payor mix may have also contributed to this limitation. The study was limited by a substantial number of patients lost to follow-up and by the limitations of retrospective chart review, which made it difficult to determine whether the patient received timely care outside our institution, leading to uncertainty about outcomes for these patients. Not all patients with a newly diagnosed mass or suspected cancer were referred to the UMC, and emergency clinicians may have known if a patient was out of network for follow-up, which could have biased who was referred to the UMC. Age, comorbidities, shared decision-making about the findings from the ED encounter, access to timely primary care follow-up, and residential distance from Mayo Clinic may have also influenced whether the patient was referred to the UMC.

The UMC’s scope of practice includes any soft tissue, intraabdominal/retroperitoneal, and lymphatic masses without association to any single organ. Patients with masses associated with one organ are referred to already established pathways at Mayo Clinic (eg, patients with lung masses are referred to pulmonary medicine). These data may not be included in our analysis. Lastly, We collected our data shortly after the last patient was enrolled. Patients with earlier referrals to the UMC had more time for follow-up, treatment, and disease progression compared to those with referrals closer to when the data were abstracted.

While it would be instructive to know the total number of patients with incidental masses identified during the study period, this information was not available because of several limitations. To obtain an accurate assessment of the number of people who could potentially qualify for the mass clinic, we would need to have examined a convenience sample of patients ≥16 years of age at Mayo Clinic Florida, who had a concerning mass on CT and otherwise met criteria for referral to the mass clinic. However, other factors would invariably have been considered by the emergency clinician, which are difficult to quantify from a chart review study such as the location of the mass and whether it was new or known, etc. In addition, abstracting data from radiology reports will not accurately identify all patients with a “mass,” given the different words used to describe a “mass” such as “nodule,” “hypodensity,” “hyperdensity,” “enlarged lymph node,” “lymphadenopathy,” and “cyst.” Furthermore, keyword searches are often inaccurate for characterizing the radiology report findings as new, old, present, absent, or possible.

## CONCLUSION

Patients who present to the ED with new solid tumors that do not meet admission criteria require expedited transitions of care to the appropriate specialist to avoid delays in diagnosis and treatment. We describe an ED referral process to an undiagnosed mass clinic, which can aid in triaging and examining patients expeditiously with undiagnosed masses before they are ultimately referred to the appropriate subspecialist once diagnosis is established.

Patients with incidental intraabdominal and intracranial masses have a high likelihood of malignancy. Emergency department patients are more likely to have an incidental finding of a malignancy as opposed to other patient populations, which is likely due to higher rates of advanced imaging use in the ED. Further research and quality improvement should aim to assess similar transitions of care for minority, Medicaid, and underserved populations.

## Figures and Tables

**Table:** Characteristics of emergency department patients referred to an undiagnosed mass clinic (UMC).

ED chief complaint	No. of times the ED chief complaint was listed^a^	Had a UMC appointment^a^	Cancer diagnosis^b^	Patient deceased?^b^
Abdominal pain	36	24	Yes: 13 Unsure: 3 No: 8	Yes: 4 Unsure: 6 No: 14
Chest pain or chest wall pain	12	7	Yes: 1 Unsure: 1 No: 5	Yes: 0 Unsure: 2 No: 5
Back pain	7	4	Yes: 2 Unsure: 0 No: 2	Yes: 0 Unsure: 1 No: 3
Shortness of breath	7	4	Yes: 3 Unsure: 1 No: 0	Yes: 1 Unsure: 2 No: 1
Cough	7	1	Yes: 0 Unsure: 0 No: 1	Yes: 0 Unsure: 1 No: 0
Nausea	10	4	Yes: 3 Unsure: 0 No: 1	Yes: 1 Unsure: 0 No: 3
Vomiting	3	3	Yes: 2 Unsure: 0 No:1	Yes: 1 Unsure: 1 No: 1
Abnormal test or laboratory result	3	2	Yes: 0 Unsure: 0 No: 2	Yes: 0 Unsure: 0 No: 2
Dysphagia	2	2	Yes: 2 Unsure: 0 No: 0	Yes: 1 Unsure: 0 No: 1
Loss of appetite	1	1	Yes: 1 Unsure: 0 No: 0	Yes: 1 Unsure: 0 No: 0
Diarrhea	5	2	Yes: 2 Unsure: 0 No: 0	Yes: 0 Unsure: 0 No: 2
Fall	4	1	Yes: 0 Unsure: 0 No: 1	Yes: 0 Unsure: 0 No: 1
Weight loss	3	2	Yes: 1 Unsure: 1 No: 0	Yes: 1 Unsure: 1 No: 0
Fever or chills	3	1	Yes: 0 Unsure: 0 No: 1	Yes: 0 Unsure: 1 No: 0
Constipation	3	2	Yes: 2 Unsure: 0 No: 0	Yes: 0 Unsure: 1 No: 1
“Dysuria,” “urine,” “urinating,” or “urinary” in the name of the complaint	5	3	Yes: 2 Unsure: 0 No: 1	Yes: 0 Unsure: 0 No: 3
Altered mental status	1	1	Yes: 0 Unsure: 1 No: 0	Yes: 1 Unsure: 0 No: 0
Flank pain	4	2	Yes: 1 Unsure: 1 No: 0	Yes: 2 Unsure: 0 No: 0
Mass	4	4	Yes: 2 Unsure: 0 No: 2	Yes: 1 Unsure: 0 No: 3
Neck pain	6	5	Yes: 2 Unsure: 1 No: 2	Yes: 1 Unsure: 1 No: 4
Fatigue	4	3	Yes: 2 Unsure: 1 No: 0	Yes: 1 Unsure: 1 No: 1
Swollen glands	1	0	NA	NA
Weakness	4	1	Yes: 0 Unsure: 1 No: 0	Yes: 0 Unsure: 1 No: 0
Sore throat	3	0	NA	NA
Night sweats	1	0	NA	NA
Myalgia	1	0	NA	NA
Headache, joint, groin, or extremity pain or swelling	25	14	Yes: 8 Unsure: 1 No: 5	Yes: 4 Unsure: 3 No: 7
Rectal pain	1	1	Yes: 1 Unsure: 0 No: 0	Yes: 0 Unsure: 0 No:1
Hypertension	4	3	Yes: 1 Unsure: 0 No: 2	Yes: 1 Unsure: 1 No: 1
Other	10	5	Yes: 2 Unsure: 2 No: 1	Yes: 2 Unsure: 1 No: 2

a Patients may have more than one chief complaint documented.

b Among those who had a UMC appointment.

*ED*, emergency department; *NA*, not applicable; *UMC*, undiagnosed mass clinic.
